# A case of solitary metastatic colon adenocarcinoma of the sternum: an unusual metastatic site

**DOI:** 10.1093/jscr/rjae656

**Published:** 2024-10-17

**Authors:** Elias Edward Lahham, Jamal Alddin Bilal Mohammad Al-Sa'ed, Mosab Mohammed Saleh Azzam, Ali Khalid Mahmoud Abu Warda, Hisham Al Amleh

**Affiliations:** Department of Radiation Oncology, Augusta Victoria Hospital, East Jerusalem, Palestine Territories, 91191, Palestine; Faculty of Medicine, Palestine Polytechnic University, P.O. Box 198, Hebron, Palestine; Faculty of Medicine, Palestine Polytechnic University, P.O. Box 198, Hebron, Palestine; Faculty of Medicine, Palestine Polytechnic University, P.O. Box 198, Hebron, Palestine; Oncology Department, Beit Jala Hospital, P.O. Box P164, Palestine

**Keywords:** colon adenocarcinoma, sternal metastasis, solitary bone metastasis, sternum malignancy

## Abstract

Colorectal cancer is a prevalent malignancy; it ranks as the third leading cause of cancer-related deaths globally. Despite the effectiveness of surgical intervention for primary tumors, ~30% of patients develop metastases, commonly in the regional lymph nodes, liver, lungs, and peritoneum. Bone metastasis is relatively rare but can occur, typically affecting vertebrae, pelvis, femur, and humerus. This study presents a 68-year-old patient with a history of locally advanced colon cancer who presented with a rapidly enlarging, painful sternal mass. Imaging and biopsy confirmed metastatic colon adenocarcinoma in the sternum. The patient was treated with radiation therapy, resulting in significant symptomatic relief and tumor reduction. This case highlights the rarity of sternal metastasis from colorectal cancer. Given the poor prognosis associated with skeletal metastases in colorectal cancer, this case emphasizes the need for vigilance in monitoring for atypical metastatic sites and the importance of tailored palliative care strategies.

## Introduction

Colorectal cancer (CRC) is one of the most prevalent cancers in the world [1], and it ranks as third in terms of cancer-related deaths in both males and females [2]. Although most of the primary CRC can be removed by surgical resection, advanced tumors sometimes show recurrences in distant organs [3]. The most popular locations of metastasis are the regional lymph nodes, liver, lungs, and peritoneum [4]. It is generally recognized that colon cancer rarely spreads to the bone [5]. Most bone metastasis occurs in vertebrae, the skull, the pelvis bone, the femur, and the humerus [5, 6]. In our case, we present a 68-year-old man who had locally advanced colon adenocarcinoma that metastasized to the sternum and discussed its presentation and management.

## Case presentation

**Figure 1:**
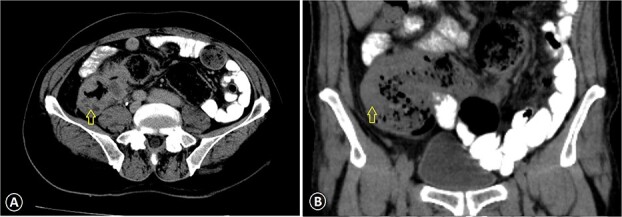
Ascending colon cancer. The CT image with IV contrast revealed a markedly thickened colonic wall (arrow) with pericolonic inflammation: (A) axial view CT scan and (B) coronal view CT scan.

A 68-year-old male patient, an ex-smoker with no significant family history of malignancy, has a known case of leukemia treated since 2018 and was diagnosed with colon cancer (pT3N1M0) in April 2023. A colonoscopy was done at the time and showed a fungating mass in the ascending colon causing significant luminal narrowing. The preoperative carcinoembryonic antigen (CEA) level was 15.3 (normal <5.0 ng/ml), and CA19-9 was 343 (normal range 0–37 units per milliliter). Multiple biopsies were taken, which confirmed invasive, moderately to poorly differentiated adenocarcinoma. A CT scan identified the mass in the ascending colon without evidence of distant metastasis (Fig. 1). The patient underwent a right hemicolectomy. Histopathological analysis of the tumor revealed invasive, moderately to poorly differentiated adenocarcinoma with focal neuroendocrine differentiation, measuring 9 cm in greatest dimension, invading the muscularis propria and peri-intestinal fat, and exhibiting numerous lymph vascular invasions with clear margins. Two out of twenty lymph nodes were positive for malignancy. Molecular profiling indicated wild-type KRAS and BRAF, deficient MMR, and negative HER-2. The tumor cells were positive for CDX2 while negative for synaptophysin. The postoperative (CEA) level was 6 ng/ml.

**Figure 2:**
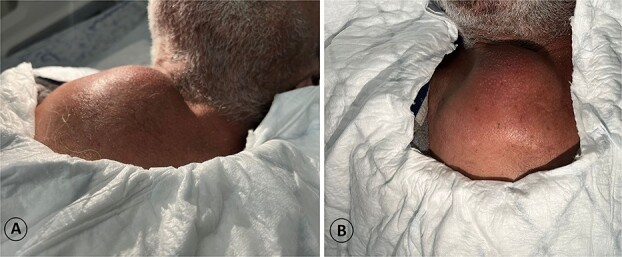
A 68-year-old male admitted for a sternal lump that developed within a few months: (A) lateral view and (B) anterior view.

**Figure 3:**
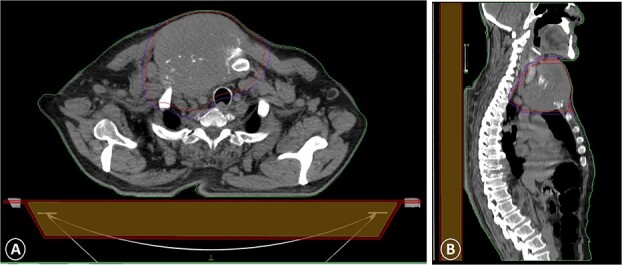
A CT scan with intravenous contrast that showed a sternal tumor of 11 by 12 cm: (A) transversal plane and (B) sagittal plane.

**Figure 4:**
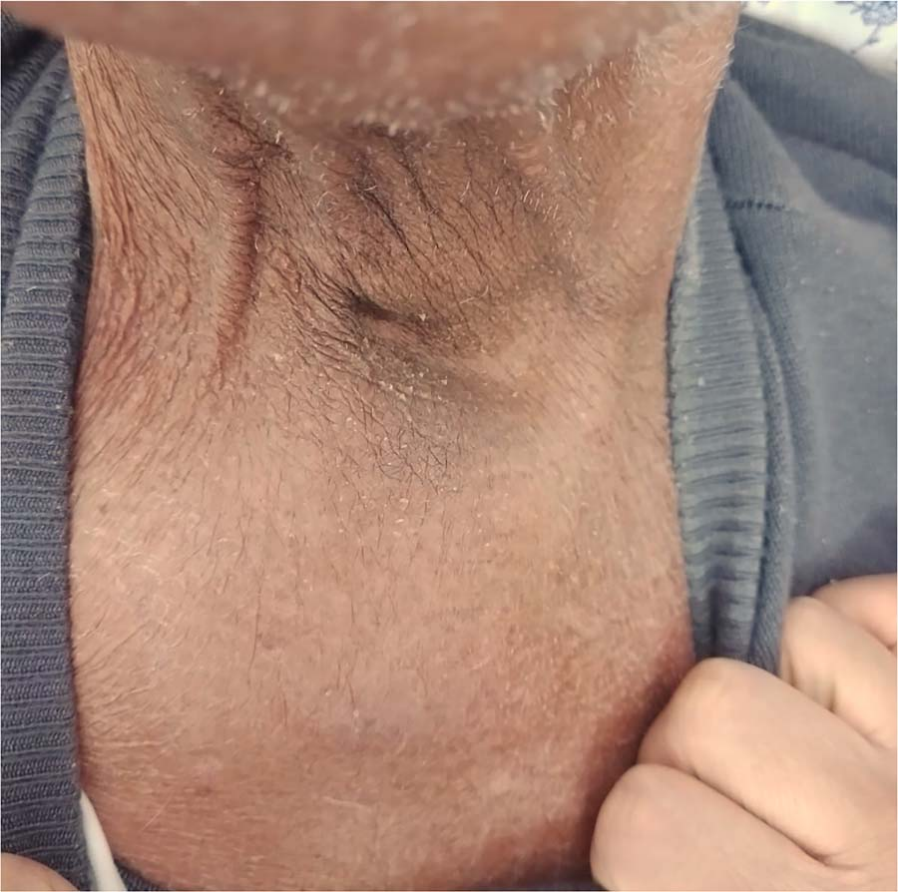
Significant clinical resolution of the mass size post radiation therapy treatment.

The patient was diagnosed with stage 3 colon cancer, and the standard of care to be given adjuvant chemotherapy was FOLFOX (leucovorin calcium, fluorouracil, and oxaliplatin) or XELOX (capecitabine ‘Xeloda’ and oxaliplatin). However, the patient has maintained on adjuvant therapy XELODA alone due to its poor performance status with comorbidities. During follow-up after the third cycle of adjuvant XELODA chemotherapy, the patient came with a rapidly enlarging, painful, hard mass on the upper part of his sternum over the last month (Fig. 2). A biopsy of the sternum confirmed metastatic adenocarcinoma consistent with the known primary colon cancer. A CT simulation was done and demonstrated a single destructive lesion in the sternum (Fig. 3). The patient received external beam radiotherapy (EBRT) to the sternum, consisting of 30 Gy delivered in 10 fractions, resulting in symptomatic improvement and a reduction in mass size post-treatment (Fig. 4). The patient was evaluated 3 months after finishing radiotherapy, with a good general condition and resolution of the sternal mass, and he is on a regular follow-up in the radiation oncology clinic.

## Discussion

CRC ranks as the third in terms of cancer-related deaths in both males and females [2]. Like the majority of other cancers, CRC has historically developed a specific metastatic pattern. The liver will be involved in ~70% of patients, making it the most frequent first site of dissemination. However, CRC metastases also affect other organs in 40%–50% of these individuals, and 20%–30% of all distant CRC metastases are found in the lung, making it the second most prevalent target organ [7]. According to reports in the literature, the incidence of bone metastases in clinical cases of colon cancer ranges from 4.7% to 10.9% [8], and in our case, the metastasis developed to the sternal bone.

The development of metastases in distant organs and the dissemination of cancerous cells beyond their original site are the outcomes of a sequence of events known as cancer metastasis. The invasion of the surrounding normal stroma marks the start of bone metastasis. The invading cells travel to the bone after separating from the original lesion. There are four ways that cancer cells might enter the bone. (i) tumor cells enter the liver via the portal vein, travel to the lungs, and then disperse throughout the arterial tree; (ii) tumor cells travel to the bone after entering the lungs via the inferior vena cava; (iii) tumor cells are dispersed along lymphatic chains before the lymphatic ducts empty into the venous system; and (iv) according to Batson, tumor cells enter the bone directly through the vertebral vein system, which consists of intraosseous vertebral veins that are located inside each vertebra, postvertebral veins, prevertebral veins, and internal vertebral veins that form a longitudinal anastomotic plexus [8].

The patient came with a rapidly enlarging, painful, hard mass on the upper part of his sternum; a CT scan was performed [9], and it showed a sternal destructive lesion. The role of biopsy is significant in the investigation of a recently discovered sternal tumor.

So the biopsy was performed and confirmed metastatic adenocarcinoma consistent with the known colorectal primary [9, 10].

For the treatment of painful, simple bone metastases, EBRT has been and still is the gold standard. While different fractionation techniques can yield high palliation rates, many prospective randomized trials have demonstrated that different fractionation strategies can yield good rates of palliation. Specifically, 30 Gy in 10 fractions, 24 Gy in 6 fractions, 20 Gy in 5 fractions, or 8 Gy in a single fraction have all been found to be effective [11]. Our patient received 30 Gy delivered in 10 fractions, which resulted in symptomatic improvement and a reduction in mass size post-treatment.

Patients with rectal malignancies that spread to the skeleton have a poor prognosis; the median survival is <10 months, and the average 5-year survival rate is 8.1% [12]. Additionally, it has been found that the prognosis for individuals with solitary metastases from colorectal tumors is worse when the metastasis includes the brain and bone, intermediate when it involves the liver, and best when it involves the lungs [13].

## Conclusion

This case shows the rarity and clinical significance of bone metastasis in CRC, specifically highlighting the unusual occurrence of sternal metastasis. Despite the standard metastatic patterns of CRC, including liver, lung, and peritoneum, this case illustrates that bone involvement, though infrequent, can occur and present with significant clinical challenges.

We recommend that early screening and diagnosis are important to facilitate treatment and prevent complications; a biopsy is critical for adequate diagnosis of a sternal tumor; and regular patient follow-up is necessary to ensure that the treatment plan is proceeding correctly.

## Data Availability

The data used to support the findings of this study are included within the article.
